# Estimation of Intercepted Solar Radiation and Stem Water Potential in a Table Grape Vineyard Covered by Plastic Film Using Sentinel-2 Data: A Comparison of OLS-, MLR-, and ML-Based Methods

**DOI:** 10.3390/plants13091203

**Published:** 2024-04-25

**Authors:** Alessandro Farbo, Nicola Gerardo Trombetta, Laura de Palma, Enrico Borgogno-Mondino

**Affiliations:** 1 Department of Agricultural, Forestry and Food Sciences (DISAFA), University of Turin, Largo P. Braccini 2, 10095 Grugliasco, Italy; alessandro.farbo@unito.it (A.F.); enrico.borgogno@unito.it (E.B.-M.); 2 Department of Science of Agriculture, Food, Natural Resources and Engineering (DAFNE), University of Foggia, Via Napoli 25, 71122 Foggia, Italy; nicola.trombetta@unifg.it

**Keywords:** protected cultivation, plastic sheet covering, precision viticulture, stem water potential, intercepted solar radiation

## Abstract

In the framework of precision viticulture, satellite data have been demonstrated to significantly support many tasks. Specifically, they enable the rapid, large-scale estimation of some viticultural parameters like vine stem water potential (Ψstem) and intercepted solar radiation (ISR) that traditionally require time-consuming ground surveys. The practice of covering table grape vineyards with plastic films introduces an additional challenge for estimation, potentially affecting vine spectral responses and, consequently, the accuracy of estimations from satellites. This study aimed to address these challenges with a special focus on the exploitation of Sentinel-2 Level 2A and meteorological data to monitor a plastic-covered vineyard in Southern Italy. Estimates of Ψstem and ISR were obtained using different algorithms, namely, Ordinary Least Square (OLS), Multivariate Linear Regression (MLR), and machine learning (ML) techniques, which rely on Random Forest Regression, Support Vector Regression, and Partial Least Squares. The results proved that, despite the potential spectral interference from the plastic coverings, ISR and Ψstem can be locally estimated with a satisfying accuracy. In particular, (i) the OLS regression-based approach showed a good performance in providing accurate ISR estimates using the near-infrared spectral bands (RMSE < 8%), and (ii) the MLR and ML algorithms could estimate both the ISR and vine water status with a higher accuracy (RMSE < 7 for ISR and RMSE < 0.14 MPa for Ψstem). These results encourage the adoption of medium–high resolution multispectral satellite imagery for deriving satisfying estimates of key crop parameters even in anomalous situations like the ones where plastic films cover the monitored vineyard, thus marking a significant advancement in precision viticulture.

## 1. Introduction

Grapevine (*Vitis vinifera* L.) cultivation has a significant socioeconomic importance around the world, with grapes being the third most popular fresh fruit after bananas and apples, with an estimated total production of about 80 million tons in 2022; around 40% of this total amount (31.5 million tons) consists of table grapes. Italy is recognized as a leading viticultural country, producing about 8.1 million tons of grapes per year [1], 1 million tons of which are table grapes. Viticulture is a highly competitive sector, requires high inputs into the production process to achieve an adequate profitability, and has to face challenges concerning the social demand for environmentally friendly agricultural management [2,3].

Precision agriculture (PA) and, more specifically, precision viticulture (PV) techniques can aid in reducing the environmental impacts of grapevine cultivation by optimizing the agronomic practices and, in particular, the use of natural resources, such as water, and inputs, such as fertilizers and pesticides [3,4,5]. PA and PV are increasingly dependent on remote sensing technologies, including satellite constellations and drone-aircraft imagery [3].

Numerous studies have shown that remote sensing data can be used to monitor key crop parameters like stem water potential (Ψstem) [6,7], leaf area index [8,9], crop coefficients [10,11], leaf nitrogen content [12,13], and crop phenology [14,15]. Often, single spectral bands or indices alone are insufficient to accurately describe the parameters under study, necessitating the use of complex models that consider parts or the entirety of the crop’s spectral signature along with the indices [6,12,13,16,17,18]. This approach can lead to severe collinearity problems, which can significantly affect the estimates [19,20]. Multivariate Linear Regression (MLR) and machine learning (ML) techniques are beneficial for complex tasks requiring multiple features [20,21,22,23]. It has been demonstrated that MLR and ML applications can effectively estimate and forecast key crop parameters [16,17,24,25].

Table grapes are a very delicate produce; therefore, their cultivation increasingly requires protecting the vineyards with plastic sheets to preserve the vegetation and bunches from external agents and condition the microclimate. This technique also allows growers to extend the harvest period by advancing or delaying the grape ripening, and to improve grape quality [26,27,28]. This practice introduces new technical challenges for PA due to the effects of plastic sheets on absorbed, transmitted, and reflected radiation, thus altering the vines’ spectral signatures [29,30,31]. This phenomenon is peculiar to the radiometric and material characteristics of plastic coverings, but could also depend on the cleanliness of the plastic film and local agricultural practices.

Changing the crop’s spectral signature can affect PA applications such as those aimed at monitoring vegetation health and growth, predicting yield, and providing variable rate irrigation and fertilization [29,32], thereby impacting resource optimization and decision-making processes.

In this context, assessing crop parameters in vineyards covered with plastic sheets for PA applications is more complex and is a quite unexplored topic. The present work aimed to help fill this this gap by monitoring a plastic sheet-covered table grape vineyard of cv Luisa, a new Italian seedless cultivar obtained and selected for in Italy (Apulia Region), using Sentinel-2 (S2) data to estimate two key crop ecophysiological parameters: (i) the intercepted solar radiation, which is closely related to foliage biomass, and (ii) the stem water potential, which is related to the water status of vines. For this purpose, statistical (Ordinary Least Squares (OLS) and Multiple Linear Regression (MLR)) and machine learning (ML) approaches were applied.

## 2. Materials and Methods

### 2.1. Study Area

**Figure 1:**
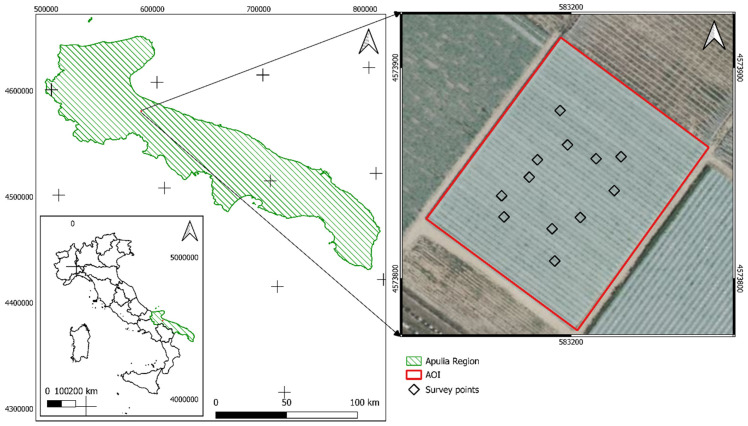
Test vineyard located in Southern Italy, Apulia Region (area with green lines). Black squares represent the spatial distribution of surveyed replicates (a square of 4 contiguous vines/replicate). The reference system is WGS 84/UTM 32N, EPSG:32633.

The study was conducted in 2022 in an adult one-hectare commercial table grape vineyard located in south-east Italy (Apulia Region, BT Province, Laporta farm, 41°31116.800′ N, 15°99390.100′ E, 66 m a.s.l.) (Figure 1). The area has a warm, temperate climate labeled as Csa (hot and dry summer Mediterranean conditions) according to the Köppen–Geiger classification [33]. The local soil presents a loamy–sandy texture. Vines (cv Luisa grafted onto 140 Ru rootstock, 2.4 × 2.4 m apart) were trained using an overhead trellis system (Tendone) pruned to 4–6 canes per vine (~10 buds/cane). Standard viticultural practices were applied in the vineyard, including leaf thinning (two interventions) and drip irrigation (1830 m^3^/year as total amount of delivered water).

**Figure 2:**
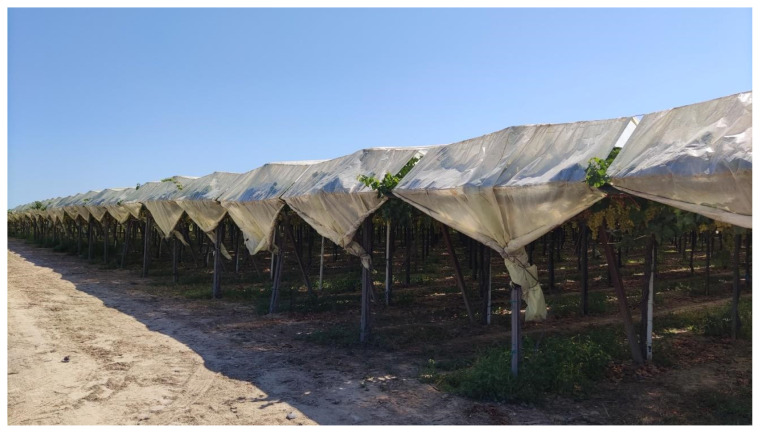
Test vineyard covered with plastic sheets.

The vineyard was covered from April to November with a commercial plastic film made of 200 μm thick low-density polyethylene, which is transparent to solar radiation. The plastic sheets were placed at a height of 3.0 m above the ground level, just above the vine canopy, following the vineyard rows; between adjacent sheets, a 20–30 cm space was left to favor air circulation (Figure 2).

### 2.2. Ground Data

Air temperature within the vineyard was automatically measured at 15 min time intervals. The sensor was positioned in the place of a missing vine at a height of 2 m above the ground (weather station provided by Horta s.r.l., Piacenza, Italy). Daily mean air temperatures were utilized to calculate the growing degree days (GDD, Equation (1)) [34].

(1) $$GDD(n)=\sum_{i=0}^{n}Max(T_{m}\left(i\right)-T_{base},\;0),$$

where *n* is the number of days from April 1st to October 31st, Tm is the average daily air temperature (°C), and Tbase is the base temperature, i.e., the minimum temperature for vegetative growth, which was assumed equal to 10 °C for grapevines [35].

Twelve replicates, each consisting of a square of 4 contiguous vines (Figure 1), were considered in order to monitor the phenological stages and assess two ecophysiological parameters: intercepted solar radiation and vine water status. Grapevine phenology was monitored during the entire growing cycle, approximately once a week, according to codes and description of the extended BBCH scale [36] (Table 1).

The intercepted solar radiation (ISR) was assessed as a parameter related to the vine vegetative growth. Data were collected from April 29th to August 22nd, on clear-sky days, at intervals of approximately 10 days. ISR was obtained by measuring, at solar zenith, the flux density of the photosynthetically active radiation (PAR, 400–700 nm, μmol photons m^−2^ s^−1^) available over the canopy (3 readings/replicate) and under the canopy (6 readings/replicate), using a solar bar (AccuPAR model LP-80 PAR/LAI, Decagon Devices, Pullman, WA, USA) [37]. Mean values and the corresponding ISR percentages were computed according to Equation (2) [37]:

ISR = [1 − (PAR_uc_/PAR_oc_)] × 100, (2)

where ISR is the percentage of intercepted solar radiation, PAR_uc_ is the flux of photosynthetically active radiation under the canopy, and PAR_oc_ is the flux of PAR over the canopy.

The vine water status was evaluated through measurements of stem water potential (Ψstem) [38] using a pressure chamber (model 3005, Soilmoisture Equip. Corp., Santa Barbara, CA, USA). Data were collected from May 25 to August 22, approximately once a week. Two completely developed leaves per replicate were sampled from the lower-internal part of the canopy. Before the Ψstem measurements, sampled leaves were enclosed in two-layer bags (plastic inside and aluminum outside) for 2 h to reach equilibrium. Measurements were performed within about 1 h (12:30 to 13:30) to keep the environmental conditions as stable as possible.

Ground measures were georeferenced using a GNSS receiver (Leica 1200, Leica Geosystem AG, Heerbrugg, Switzerland). The survey was conducted in the NRTK (Network Real Time Kinematic) VRS mode using the Puglia Region correction service (http://gps.sit.puglia.it/SpiderWeb/frmIndex.aspx, accessed on 21 June 2022). The average accuracy (3D) was about 3 cm [39].

### 2.3. Satellite Data

Earth Observation (EO) satellite imagery is presently widely available. Nevertheless, not all images are suitable for applications in precision agriculture (PA). PA, in fact, shows some specific requirements: (1) the images’ geometric resolution must be consistent with the size of monitored fields; (2) the temporal resolution has to be sufficiently high to significantly track the phenological stages of crops; (3) the spectral bands should be sensitive to key crop parameters such as biomass, photosynthetic activity, and leaf water content; (4) the image cost has to be consistent with the ordinary income of the agronomic sector (possibly free); and (5) the data have to be provided in a ready-to-use and harmonized form.

With respect to these constraints, S2 data from the EU Copernicus Program are suitable. The S2 MSI (Multi spectral Instrument) sensor acquires 13 bands in the range of 400–2500 nm with a nominal temporal resolution of 5 days and a maximum spatial resolution of 10 m. Additionally, S2 images are provided free of charge and, if obtained with a processing level of 2A, are already ortho-projected and BOA (Bottom of the Atmosphere)-calibrated.

The S2 images used in this work were obtained through the Google Earth Engine (GEE) platform from the S2 Harmonized Level 2A Collection. The declared spatial accuracy of the data is 3 m and the radiometric resolution is 12 bits [40] (Table 2).

According to the literature, single bands and several spectral indices [3] can be used as predictors for ISR and Ψstem. All S2 bands (excluding B1, B9, and B10) were used in this work. 

In the meantime, some vegetation indices were computed as well (Equations (3)–(10)).

NDVI = (B8 − B4)/(B8 + B4)(3)

EVI = 2.5∙(B8 − B4)/(B8 + 6∙B4 − 7.5∙B2 + 1)(4)

GNDVI = (B8 − B3)/(B8 + B3)(5)

NDRE = (B8 − B5)/(B8 + B5)(6)

NDWI_1_ = (B8 − B12)/(B8 + B12) (7)

NDWI_2_ = (B3 − B11)/(B3 + B11) (8)

NDWI_3_ = B11/B12 (9)

NDMI = (B8 − B11)/(B8 + B11)(10)

where B2, B3, B4, B5, B8, B11, and B12 correspond to the spectral ranges reported in Table 2. Bands showing a GSD = 20 m were oversampled to 10 m (nearest neighbor).

NDVI, EVI, and GNDVI are spectral indices widely used for estimating crop biomass [42], assessing plant health status [43,44], and conducting phenological analyses [45,46]. Conversely, NDWI_1-2-3_ and NDMI are primarily related to water content and can be used for Ψstem prediction [47,48,49]. No soil-adjusted spectral indices (such as SAVI, OSAVI, MSAVI, or MSAVI2) were considered in this study due to the specific nature of the vineyard. In fact, the Tendone training system resulted in a high soil coverage (Figure 2), eliminating the need for spectral indices designed to reduce the soil effect in the vegetation’s spectral response. Furthermore, Borgogno et al. [29] identified a strong correlation between the NDVI and MSAVI2 spectral indices in vineyards covered with plastic sheets, suggesting that these two indices provide similar information.

Spectral bands and spectral indices time series (TS) were generated using GEE. The local (pixel-level) temporal profile of the index/band was initially filtered using the Scene Classification Layer (SCL) provided with the image, by masking out the observations labeled as clouds, shadows, and cirrus. It was finally regularized at a 1-day time step with 1st-order linear interpolation [50] in order to ensure a satellite observation (even if estimated) for all the dates of the ground surveys (Table 3).

On each sampling date, the corresponding spectral band or index was extracted. The mean value of the pixels underlying the ground samples (usually one or two pixels per sampling area) was computed for that specific date. This process resulted in the extraction of the mean values of indices/bands from the regularized image TS. The values were then incorporated as new attributes into the vector layer that identifies the locations of the ground samples.

Based on a previous work [29], a preliminary analysis was conducted to investigate the contribution of the plastic film to the spectral responses of the vines during the 2022 growing season. Eighteen spectral features were considered during the analysis (Table 4). For this purpose, a change point analysis was performed with reference to the field-average daily interpolated TS of the above mentioned 18 spectral features. Specifically, the Pettit’s non-parametric test was used to locate the strongest breakpoint along the time series using two subsequent windows with a size equal to 20 days [51].

### 2.4. Relating ISR and Ψstem with Spectral and Meteorological Features

ISR/Ψstem estimates were obtained according to 3 different approaches: (i) 1st order Ordinary Least Square regression (OLS), (ii) Multiple Regression (MLR), and (iii) machine learning.

As proposed by previous studies, crop physiological parameters can be estimated from spectral features by incorporating machine learning algorithms in the process [16,52]. No Deep Learning (DL) approach have been employed due to the limited amount of training data and the consequent high risk of model overfitting [53,54].

In line with best practices, OLS, MLR, ML, and DL models should be used in their most straightforward form, avoiding unnecessary complexity that could lead to overfitting and poor generalization to new data [55,56]. The goal is to achieve the expected results with the simplest model. ML and MLR algorithms, theoretically, can include all available features (predictors); however, this can lead to unnecessarily large models. To tackle this problem, in this work, a preliminary step was performed to select the most meaningful features and to tune the model parameters. This procedure was carried out on the same dataset, both excluding and including the GDD data derived from the meteorological station located beneath the plastic sheet as a possible available variable in the feature selection process. This approach was chosen to test possible approaches that do not have to rely on the meteorological data from under the plastic sheets, which are not always accessible.

A k-fold cross-validation strategy (k = 5) was utilized to mitigate overfitting and to estimate the accuracy of the models. This strategy was adopted during the training and testing phases for the single feature OLS, MLR, and ML models. Specifically, all testing folds were combined to recreate the entire test dataset and obtain the general evaluation metrics reported in Section 2.4.4.

#### 2.4.1. Polynomial Regression

To investigate the relationship between spectral features and ISR/Ψstem, first-order polynomial OLS regressions were used [57], as shown in Equation (11):

(11) y=a·x+b

where y corresponds to the dependent variable (i.e., ISR or Ψstem), x is the independent variable (i.e., spectral or meteorological feature), and a and b are the slope and intercept model parameters, respectively.

Conversely, the relationship between GDD and active biomass (for which ISR is a proxy) is known to be well modeled by a 2nd-order polynomial regression (i.e., a concave parabola) [29,45]. Therefore, the relationship between ISR and GDD was modeled according to Equation (12):

(12) $$y=a\cdot x^{2}+b\cdot x+c$$

where y corresponds to the dependent variable (i.e., ISR), x is the independent variable (i.e., GDD), and a, b, and c are the model parameters.

The coefficients for the models were obtained using the entire dataset for training, with the aim of highlighting patterns in the data. Conversely, the evaluation metrics reported in Section 2.4.4 were computed for the test dataset. This dataset was derived from the cross-validation procedure, during which, a different polynomial regression was fitted for each training fold. This dual approach enables a comprehensive exploration of the relationships between the spectral–meteorological features and the two ecophysiological ones. At the same time, it allows for a thorough testing of the models using the entire dataset, thereby minimizing the risk of overfitting.

#### 2.4.2. Multiple Linear Regression

Multiple linear regression (MLR) with all the variables was used to predict ISR/Ψstem. Performing MLR considering several variables might be detrimental due to the risk of collinearity between them, causing model parameter instability [58]. This phenomenon is a common issue in multiple regression analyses, which is known as multicollinearity. To address this issue and select variables, multiple linear regression with stepwise selection (MLRS) was employed. Akaike Information Criterion (AIC) was used to select the best features that strongly contributed to the model [59]. Due to the limited number of ground samples and the need to thoroughly test the models across a wide range of cases, both MLR and MLRS were evaluated using a 5-fold cross-validation approach. Therefore, each model was effectively tested using the entire dataset.

#### 2.4.3. Machine Learning Algorithms

ML algorithms can be used to efficiently model complicated and, possibly, non-linear relationships. Specifically, Random Forest Regression (RFR), Support Vector Regression (SVR), and Partial Least Squares Regression (PLSR) were chosen for this study [60,61,62].

In order to limit the number of variables used in the ML training, a forward feature selection was utilized, calibrating the same model that is fed with an increasing number of features to look for a significant reduction in RMSE.

SVR and RFR require an additional step aimed at identifying the optimal hyperparameters; this was achieved through a GridSearch approach that was repeated for every feature group used during the forward feature selection step. Table 5 reports the parameters values tested in this step. All the features used for ML training were normalized a priori to avoid any scale-related problems. This iterative approach led to the identification of the best model configuration for the 3 tested ML algorithms. Finally, the best model for each of the considered algorithms: PLSR, SVR, and RFR. Similar to Section 2.4.2, the ML training and testing were performed using a 5-fold cross-validation approach. All the ML analyses were conducted using Python 3.10.

#### 2.4.4. Model Performance Evaluation

All models were assessed based on the relationship between observed and predicted values. The *p*-value was the first parameter considered to evaluate the significance of the relationship between dependent and independent variables for the OLS, MLR, and MLRS approaches. The coefficient of determination (R^2^, Equation (13)) and the Root Mean Squared Error (RMSE, Equation (14)) were chosen as performance indicators for all models [63]. Additional insights were obtained through the analysis of the slope and intercept of the first-order linear model for the observed and predicted values of the dependent variable (O-P slope and O-P intercept, respectively). Statistical analyses were conducted using R software, version 4.3.1 [64].

(13) $$R^{2}=1-\frac{{\sum_{i=1}^{n}\left(y_{i}-\hat{y_{i}}\right)}^{2}}{{\sum_{i=1}^{n}\left(y_{i}-\underline{y_{i}}\right)}^{2}}$$

(14) $$RMSE=\sqrt{\frac{{\sum_{i=1}^{n}\left(y_{i}-\hat{y_{i}}\right)}^{2}}{n}}$$

where $y_{i}$ and $\hat{y_{i}}$ are the *i*-th measurement and corresponding predicted value for i = {1, …, n}, n is the number of measurements, and $\underline{y_{i}}$ is the mean of all the values.

The RMSE, R^2^, O-P slope, and O-P intercept were computed using the test set derived from the cross-validation procedure.

## 3. Results

### 3.1. Ground Data Variability

ISR and Ψstem were measured through ground surveys 12 and 9 times, respectively. Table 6 reports the mean, minimum, maximum, and standard deviation values for both parameters. On DOY 119 (phenological stage of ‘six leaves have unfolded’), the vine shoots were long enough to guarantee a high accuracy in ISR determination. The average solar radiation intercepted by the foliage ranged from 24.86% on DOY 119 (April 29) to 87.48% on DOY 234 (August 22, ‘end of wood maturation’, and end of field measurements). The ISR values increased linearly as the shoots grew until DOY 190 (July 9): this was the moment when the veraison occurred and shoot elongation stabilized. Afterwards, due to viticultural canopy management (i.e., basal leaf removal and shoot topping), the ISR values decreased until DOY 216 (August 4, ‘berries ripe for harvest’); on DOY 234 (August 22), it reached a value close to the one on DOY 190. The minimum and maximum ISR values were found to be 18.78% (DOY 119) and 93.98% (DOY 181, ‘all berries are touching’), respectively.

The average stem water potential ranged from −0.494 MPa on DOY 162 (June 11, ‘berries beginning to touch’) to −1.094 MPa on DOY 207 (July 26, ‘berries brightening in color’). The maximum and minimum Ψstem values were found to be −0.425 MPa (DOY 145, ‘fruit is set’) and −1.335 MPa (DOY 207), respectively. According to the threshold values proposed by van Leeuwen et al. [65], the average vine water deficit was null from the beginning of the measurements until DOY 162, weak on DOYs 181 and 190, weak to moderate on DOYs 201-207, and weak again on DOYs 216 and 234, i.e., the last two measurement dates.

The two ecophysiological parameters showed considerable variability during the growing season of vines, as demonstrated by both the magnitude of the standard deviation and the extended ranges of values. This variability suggests wide spatial and temporal fluctuations, resulting in a sufficiently large dataset covering the ISR range from 18.78% to 93.98%, and Ψstem range from −0.425 MPa to −1.335 MPa, despite the fact that the data were collected from a single vineyard in just one year.

### 3.2. Temporal Trends of Vineyard Reflectance and Spectral Indices

**Figure 3:**
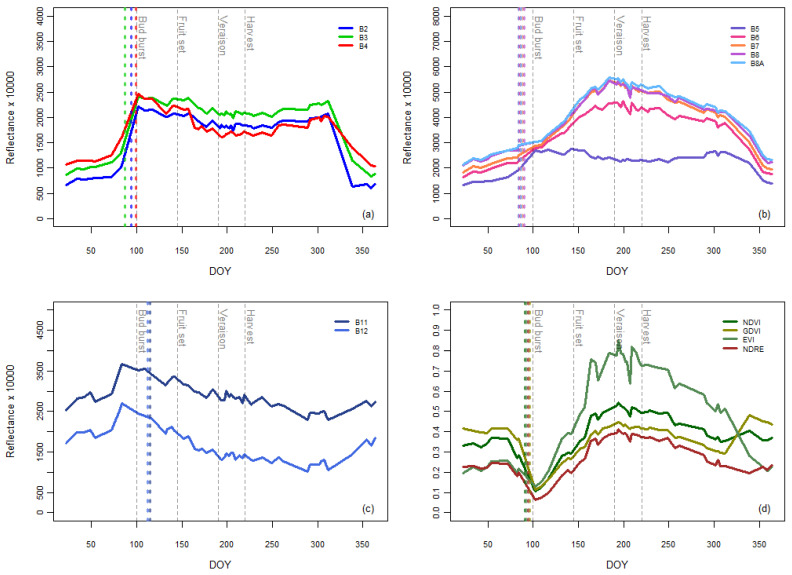
Temporal trends of satellite-derived spectral features collected during the year (DOY): (**a**) reflectance values of visible bands (B2, B3, B4); (**b**) reflectance values of red-edge and NIR bands (B5, B6, B7, B8, B8A); (**c**) reflectance values of shortwave infrared bands (B11, B12); (**d**) vegetative indices (NDVI, GDVI, EVI, NDRE). Vertical dashed lines correspond to the time series change points identified by the Pettit test (dashed line colors match the temporal profile colors).

Some preliminary analyses explored the contribution of the plastic film to the vineyard’s spectral behavior. Both single spectral bands and spectral indices, namely vegetation indices (VIs), were considered (Figure 3). Starting from the beginning of the year, the S2 reflectance of bands B2, B3, B4, and B5 showed a rapid increase at DOY 100, which coincided with budbreak, i.e., the time when the plastic sheets were un-rolled over the vineyard, and decreased rapidly in early December, when the plastic sheets were rolled up. As for the other bands, the B11 and B12 reflectance values increased before budbreak and again at the beginning of December, while the spectral response of B6, B7, B8, and B8a seemed to not be directly affected by the film handling.

These observations, utilizing the abrupt transitions between absence/presence/absence of the plastic covering during the vine annual cycle, align with findings by Borgogno et al. [29], who directly compared reflectance values coming from covered and uncovered vineyards. Those authors noted that bands B7, B8, and B8A showed no significant difference between covered and uncovered vineyards, which was different from the results from the visible and SWIR bands.

As far as indices are concerned, Figure 3 shows notable reductions in VI around DOY 100 (when plastic sheets were un-rolled over the vineyard). This can be related to a significant increase in reflectance values of visible bands and B5. In contrast, no analogous increase was found for B8; this suggests an apparent loss of photosynthetic activity while maintaining the same biomass (i.e., reduction in vegetative indices). Conversely, in early December, when the plastic sheets were rolled up, a significant increase in NDVI and GNDVI values were noted, together with a decrease in EVI values. The seemingly stable trend of the NDRE values may be explained by the combination of bands B5 and B8, which both showed gradual changes towards the end of the season (when the plastic sheets were rolled up). VI also showed similar patterns from budding until after harvesting. According to these findings, and considering that it is the most used index for monitoring grapevine behavior during the growing season, NDVI was selected as the reference index [66]. Despite the presence of the plastic film from budbreak until after harvesting (when the onset of leaf senescence took place), NDVI showed a pattern that was quite consistent with the seasonal trends observed in an uncovered vineyard [67]. The minimum NDVI values were recorded in April. Conversely, the NDVI maxima were reached in July (shortly after veraison), which were followed by a general decrease in August. It would seem that NDVI trends could be used to efficiently monitor vines in spite of the presence of this type of plastic cover. This finding is aligned with a previous work [29].

Change point analyses enable the easy identification of the most abrupt changes in each TS. Figure 3 presents the identified change points for each TS. The analysis clearly identified the moment of plastic sheet opening as the most significant for all the spectral bands and indices (*p*-values < 0.0001), thereby supporting all the previous analyses. Interestingly, the most abrupt changes were identified when the plastic sheets were opened on the field rather than when they were closed.

### 3.3. Relating ISR and Ψstem with Spectral and Meteorological Features: Linear Regression Analysis

#### 3.3.1. ISR Estimation

Given the results of the preliminary analysis (Figure 3), first-order polynomial linear relationship models were tested to relate ISR with the S2 bands and spectral indices. Table 7 reports the values of the computed intercept, slope, *p*-value, R^2^, RSME, and coefficients derived from the fitted first-order polynomial model between the observed and predicted values (O-P intercept and O-P slope). All the satellite spectral features showed significant relationships with ISR (*p*-value < 0.0001). The only exception was NDWI_2_.

The most significant bands (*p*-value < 0.0001 and R^2^ > 0.80) were found to be those located between 740 and 865 nm (Table 2). In particular, B6, B7 (red edge), B8, and B8a (NIR) proved to be the best predictors with an RMSE value of about 7%. Additionally, the linear model for the observed and predicted values showed that B7 and B8A led to the most aligned predictions (i.e., O-P intercept < 7; O-P slope = 0.9).

Regarding the vegetation indices, the lowest errors were achieved with NDRE (R^2^ = 0.872 and RMSE = 7.82%). Among the water indices, the strongest correlation was found with NDMI and NDWI_1_ (see Equations (7) and (10)). Conversely, NDWI_2_ and NDWI_3_ resulted in the weakest relationships. In general, the vegetation indices (i.e., NDVI, NDRE, GNDVI, and EVI) and NDMI led to a minor overestimation of ISR at low values (O-P intercept < 10). Simultaneously, a fairly linear relationship was maintained between the observed and predicted ISR, with an O-P slope greater than 0.85.

The relationship between ISR and GDD was modeled with a second-order polynomial model, as suggested by previous studies [29,45]. The relationship between the environmental variable GDD and ISR had the highest R^2^ and the lowest RMSE values (0.917 and 6.31%, respectively). The intercept derived from the first-order linear model for the observed and predicted ISR values was the smallest compared to those obtained from the spectral features (O-P intercept = 5.53). In contrast, the O-P slope was the highest at 0.91.

#### 3.3.2. Ψstem Estimation

Table 8 shows the Ψstem estimation results from the univariate OLS analysis alongside the first-order model parameters for the observed and predicted Ψstem values for all analyzed features (O-P intercept and O-P slope). The analyses resulted in low R^2^ values (<0.45) and high RMSE values (>0.17 MPa). Regressions built using B6, B7, B8, B8A, NDWI_2_, and EVI as predictors (considered individually) were significant with *p*-values > 0.0001. B3 was the best predictor, showing an R^2^ and a RMSE value of 0.418 and 0.174, respectively. Reflectance in the visible and SWIR bands was found to be positively correlated (slope > 0) with Ψstem.

The results also showed that the spectral indices performed worse compared with single bands in predicting Ψstem. The corresponding RMSE values were around 0.2 MPa. However, it can be noticed that all spectral indices had a negative correlation with Ψstem (slope < 0). The unique exception was NDWI_2_, which showed a slightly positive slope but with a very dispersed point cloud (R^2^ = 0.031). This phenomenon can be interpreted as a non-significant relationship. Additionally, the O-P slope was about 0.04, confirming the hypothesis.

Regarding the O-P parameters, it can be observed that the intercept was always negative (<0), while the slope was always smaller than 0.4 with the only exceptions being B3 and GDD (0.43 and 0.46, respectively). The O-P parameters highlight the unsatisfying predictive capabilities of the first-order linear models applied to both the spectral and meteorological variables.

### 3.4. Relating ISR and Ψstem with Spectral and Meteorological Features: Multivariate Approach

Multivariate regressions can be prone to overfitting, especially when dealing with limited datasets and a large number of independent variables. To address this, two strategies were employed: an MLR using all available features (with and without Growing Degree Days—GDD) and a Multiple Linear Regression with stepwise feature selection based on Akaike Information Criterion (MLRS) (also with and without GDD). The results of both the MLR and MLRS for ISR and Ψstem estimation are reported in Table 9 and refer to the test set derived from the 5-fold cross validation.

The MLR approach yielded satisfactory results for ISR estimation compared to the univariate models. Specifically, the RMSE and R^2^ values obtained from the 5-fold cross-validation were 7.26% and 0.88, respectively. Notably, the inclusion of the GDD variable significantly improved the results, with R^2^ increasing to 0.93 and RMSE decreasing to 5.39%. However, it is important to note that these results were achieved by considering all variables (18 without GDD and 19 with GDD).

The MLRS approach, which utilized AIC for feature selection, reduced the number of predictive variables by selecting only the most informative ones, thereby mitigating the risk of overfitting. As a result, both RMSE and R^2^ showed improvement with GDD (5.13% and 0.94, respectively) and without GDD (6.65% and 0.90, respectively). Notably, the O-P slopes and intercepts were always higher than 0.9 and lower than 6, respectively. This highlights a strong linear relationship between the observed and predicted ISR values, specifically for the MLRS when considering GDD, which showed the lowest O-P intercept value (3.05) and the highest O-P slope (0.95).

As for Ψstem estimation, the MLR approach outperformed the univariate models, yielding RMSE and R^2^ values of 0.136 MPa and 0.53, respectively, when GDD was included, and 0.134 and 0.55, respectively, when it was not. Furthermore, by selecting only the most informative variables, the RMSE decreased to 0.117 and 0.118, and the R^2^ increased to 0.62, both with and without GDD. Regarding the O-P intercepts and slopes, MLR and MLRS managed to effectively reduce the former and increase the latter, compared to the univariate models.

### 3.5. Relating ISR and Ψstem with Spectral and Meteorological Features: Machine Learning Approach

ML algorithms are renowned for being able to model complex and non-linear relationships. The results reported in the previous sections highlighted the potential and limitations of linear OLS and multivariate approaches like MLR/MLRS in predicting ISR and Ψstem for vines. For these reasons, three ML algorithms (RFR, SVR, and PLSR) were tested to model the same relationships. ML training and testing were performed with and without GDD as an additional predictor. The optimization and feature-selection step made it possible to tune the model parameters and to select the most significant features (Table 10 and Table 11). All the reported results refer to the test set derived from the 5-fold cross validation.

The ISR estimates derived from the ML algorithms demonstrated lower RMSE and higher R^2^ values compared to those obtained from linear OLS. Specifically, the lowest RMSE was achieved when RFR was used with GDD as an additional predictor, resulting in an RMSE of 4.7% with an R^2^ of 0.96. When these results were compared to those from MLRS, a few improvements were observed. Notably, the slope and intercept of the observed–predicted relationship for RFR, when GDD was considered, were the highest (0.96) and lowest recorded (1.8), respectively. Interestingly, the worst performing models were the SVR, which considered only two variables (B6 and B7), and PLSR, which also selected only two variables (B5 and B8A), which achieved average results.

Regarding Ψstem estimates, the ML approach showed lower RMSE and higher R^2^ values compared with the ones from OLS. On the other hand, when compared to MLR and MLRS, only a few improvements were noticeable. Specifically, the minimum RMSE and highest R^2^ were obtained using RFR with GDD as additional predictor (RMSE = 0.101 MPa and R^2^ = 0.81). Additionally, the O-P intercept and slope were the closest to zero and the closest to one observed so far in the Ψstem modeling task. Conversely, the worst performing models were RFR (B3, B5, B8, and NDWI) and PLSR (B3, B4, B7, B8, B8A, and NDRE) with RMSE values equal to 0.148 and 0.147 MPa, respectively.

### 3.6. ISR and Ψstem Estimation Maps

**Figure 4:**
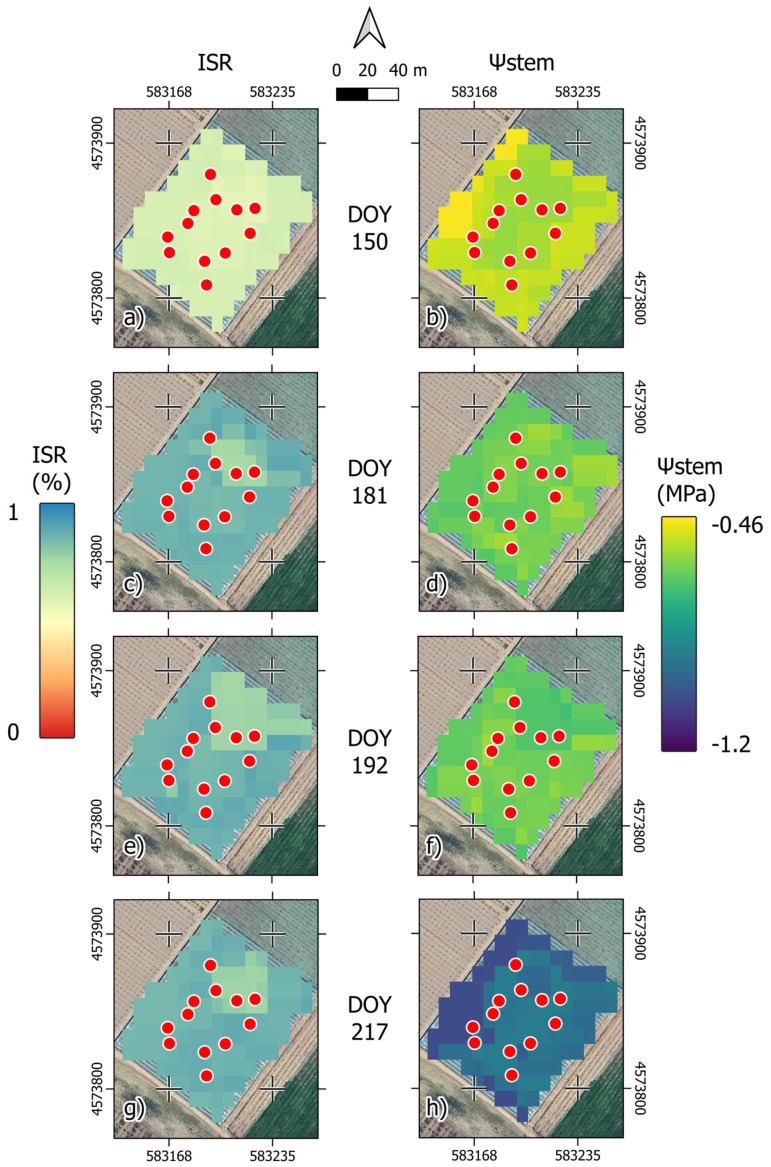
ISR (**a**,**c**,**e**,**g**) and Ψstem (**b**,**d**,**f**,**h**) prediction maps of the studied vineyard derived from the RFR models at (**a**,**b**) DOY 150 (‘berries are groat-sized’); (**c**,**d**) DOY 181 (‘all berries are touching’); (**e**,**f**) DOY 192 (‘beginning of ripening’); (**g**,**h**) DOY 217 (‘after harvest’). Red dots correspond to the ground surveyed areas. The reference system is WGS 84/UTM 32N, EPSG:32633.

The potential of this study lies in the integration of satellite data, ground surveys, and ML/MLRS models. Within this framework, the first step was to train an appropriate model to estimate vegetation parameters such as ISR/Ψstem based on ground samples. The application of precision viticulture lies in obtaining field estimates of these two parameters based on real-time satellite data. For this reason, four ISR and Ψstem estimation maps were generated using the best-performing models (RFR with GDD). Four key vineyard stages were explored: ‘berries are groat-sized’ (Figure 4a,b) at DOY 150; ‘all berries are touching’ (Figure 4c,d) at DOY 181; ‘beginning of ripening’ (Figure 4e,f) at DOY 192; and ‘after harvest’ (Figure 4g,h) at DOY 217.

The ISR maps (Figure 4a,c,e,g) showed a gradual increase in vegetation cover over time. However, in the N-E part of the field, a consistently less vegetated area was present. The differences in ISR between areas were amplified by vegetative growth, as observed in the map at DOY 192. The Ψstem values (Figure 4b,d,f,h) showed variability in both the space and time domains. At DOY 150, the outer zones showed higher Ψstem values compared to the inner zones. However, with subsequent maps (Figure 4d,f,h), this trend reversed. Furthermore, it is notable in these three maps that there was an apparent trend in the distribution of Ψstem across the vineyard, as seen in the ISR values.

## 4. Discussion

Remote sensing, and Sentinel-2 data in particular, has emerged as an alternative [32,68,69,70] for estimating vine parameters, offering advantages over traditional time-consuming techniques that are commonly employed to assess plant water status [71] and vegetative growth [72,73]. However, remote sensing-based deductions can be affected by exceptional local conditions, namely the presence of plastic coverings (or nets), that can lead to unexpected results if not properly addressed [29]. Previous works demonstrated that, despite coverings, spectral signals from vineyards are still significant especially if the plastic sheets are specifically designed for canopy protection [29]. On other hand, the radiometric characteristics of plastic sheets have an impact on transmitted radiation [30,74,75] and can have a significant effect on the reflection from the canopies. With this premise, this work intended to explore the capability of satellite-derived spectral bands and indices to estimate two crucial ground parameters: ISR and Ψstem.

Preliminary analyses were conducted to identify change points in the spectral time series. These analyses confirmed that plastic sheets cause abrupt changes in the spectral reflectance of vegetation. This highlights the need for proper models to estimate ecophysiological parameters. These models should be specifically developed for vineyards covered with plastic sheets.

ISR estimation with quadratic models applied to GDD led to the lowest overestimation at low ISR values compared to the models based on spectral bands and spectral indices. However, while GDD can effectively model ISR, it is important to note that the temperature readings were derived from a single meteorological station located beneath the plastic sheets (i.e., uniform across the entire vineyard). Therefore, this approach does not account for in-field variability.

The outcomes from the spectral linear regression analysis indicated that S2 B6, B7 (vegetation red edge), and B8-B8A (NIR) are good predictors of ISR, showing a low sensibility to plastic coverings. The importance of red-edge bands to describe vine vegetative parameters is widely acknowledged in the literature [76]. Their importance was furtherly confirmed by their inclusion in the MLRS and ML approaches designed for ISR estimation (Table 9 and Table 10). In particular, SVR provided estimates with an RMSE of 6.4%, leveraging two red edge bands, namely B6 and B7. This is particularly interesting since they are minimally influenced by plastic coverings. To substantiate this observation, additional studies on a more extensive array of plastic covers have to be realized.

As expected, reflectance values in the visible range showed a negative correlation with ISR, but for different reasons. The B2 and B4 wavelengths are known to be absorbed by active vegetation to feed their photosynthetic activity. In contrast, B3 (green)’s stronger absorbance is mainly due to leaf structures which are expected to be thicker when photosynthesis is stronger. In the visible spectrum, the highest R^2^ was found for B4 (665 nm), while for the non-visible spectrum, the highest R^2^ was found for B8A (885 nm).

Despite the single bands showing a good correlation with ISR, some further improvements are expected to come from the adoption of VI spectral indices that are known to absorb most of the residual calibration uncertainties of BOA (Bottom of the Atmosphere) reflectances (especially from film-covered vines). Films, in fact, can differently affect reflectance values depending on bands (Figure 3). In contrast, VIs generally showed a reduced difference for both covered and uncovered vines during the rolling/unrolling phases of vineyard management.

In the univariate OLS-based approach, VI and NDMI provided estimates of ISR with an approximately R^2^ of 0.86 and an RMSE of 8% for a vineyard characterized by notable spatial and temporal variability in solar radiation intercepted by the canopy. Among the VIs, the Normalized Difference Red Edge (NDRE) emerged as particularly noteworthy. NDRE was already documented as being useful in predicting ISR for maize and soybean [52]. Other authors used NDVI to monitor the effect of the vineyard leaf thinning practice with satisfactory results [77]; in this work, NDVI proved once again to be a useful and versatile index to estimate leaf coverage. Water indices have opposing behaviors depending on the inclusion or exclusion of the NIR band. The NIR band contribution in the NDWI_1_ and NDMI formulas improves the prediction of biomass traits of local vegetation. Conversely, indices where NIR is not present bands (i.e., NDWI_2_ and NDWI_3_) produce worse predictions.

Generally, the lowest RMSE was achieved when RFR was used with GDD as an additional predictor, resulting in an RMSE of 4.7% with an R^2^ of 0.96. When these results were compared to those from MLRS, a few improvements were observed. Considering these findings, it is important to discern the applicability of different modeling approaches based on the complexity of the problem. For relatively simple linear problems, such as the estimation of ISR, the OLS approach should be preferred due to its simplicity, ease of interpretation, and lower computational demand. The OLS method, despite being outperformed by the MLRS and ML algorithms in terms of RMSE, remains a robust and efficient tool for tasks where the relationships between variables are well-defined and less intricate.

Conversely, as far as Ψstem estimation is concerned, the prevailing approaches emphasize the use of more intricate modeling techniques [6,16,78]. Bands and spectral indices were revealed to be significantly correlated with Ψstem (OLS); nevertheless, the corresponding relationships showed low R^2^ values. In particular, B3 proved to be the best predictor for Ψstem (Table 8). A preliminary investigation indicated that a reduction of B3 reflectance (530–550 nm) provides a quite accurate explanation for the change in leaf water potential induced by water stress when employing hyperspectral imaging with grapevines [79]. Reflectance in the visible and SWIR bands was found to be positively correlated with Ψstem, despite increasing reflectance in the SWIR range being commonly found to be strongly correlated with a decrease in leaf water content [79]. However, a negative slope value was also found for SWIR bands in a study conducted on cotton [80]; furthermore, in grapevines, it was found that the slope of the linear model relating Ψstem with B8a and B11 can be negative for Ψstem values > −0.70 MPa and positive for Ψstem < −0.90 MPa [81]. According to our results, Ψstem values lower than −0.90 MPa were found from DOY 201 (July 20) to DOY 234 (August 22, end of the field measurement); this corresponds, in the study area, to the typically warmer summer period. In these conditions, the vines located in the portions of the vineyard with a weaker performance can suffer severely. Differences in vine biomass could explain these findings. In fact, given the vineyard variability, Ψstem corresponded to biomass. Remembering that stem water potential is an indicator of water status, whereas SWIR reflectance is an indicator of water quantity, it is possible that vines with a higher biomass, even if they are water stressed, could retain a high total water amount. In contrast, models using red-edge and NIR bands showed a lower degree of prediction capability; however, when combined with bands in the SWIR and visible spectrum, red-edge and NIR bands contributed information for the indices with more pronounced relationships with Ψstem, in accordance with other authors who demonstrated that the short-wave infrared, near-infrared, and red bands, along with their corresponding indices, are significantly associated with the water status of vines [6,52]. Additionally, these features were incorporated into the MLR, MLRS, RFR, SVR, and PLSR models (Table 11), confirming their contribution to the estimation of the plant’s water status. These improved modeling performances underscore the necessity of multiple spectral variables for accurate Ψstem estimation.

In this study, ML algorithms, specifically SVR and RFR, were shown to achieve satisfactory results, with RMSE values of 0.125 and 0.148 MPa, respectively. Similar findings were reported in a study where vine predawn water potential was predicted using hyperspectral data in two vineyards [82]; the RMSE was reported to be approximately 0.12–0.11. A more recent study applied ML to Sentinel-2 satellite data to estimate Ψstem, finding an RMSE of 0.26 [16]; this result was obtained over three growing seasons and incorporated a dataset of 348 Ψstem measurements from various cultivars.

In addition, we explored the incorporation of GDD into the models; it served to include microclimatic temperature information. The results exhibited further improvement, yielding a reduced RMSE of only 0.122 for SVR and 0.101 for RFR. This improvement is particularly remarkable when considering the variability observed in Ψstem within the vineyard on the same dates; however, incorporating GDD involves introducing a variable into the models that needs to be measured in the field. Conversely, the MLR and MLRS approaches did not benefit from the inclusion of GDD as a variable, leading to approximately the same results. The results clearly showed that the MLRS and ML models, specifically RFR, can be employed to achieve satisfactory results, outperforming the univariate OLS approach. Based on the favorable outcomes and results from prior investigations, it seems that the inclusion of a plastic film did not impede the efficacy of multivariate and machine learning models for the estimation of Ψstem.

The models relied on data with a range of Ψstem values, spanning from −0.425 MPa to −1.335 MPa. Lower Ψstem values were not measurable during the season due to irrigation support given to the table grape vineyard and, therefore, could not be included. Consequently, the efficacy of estimating water status, especially in the presence of severe water stress (<1.4 MPa), requires verification in future studies.

Additionally, other studies have proposed methodologies based on functional time series analyses to estimate vineyard variability and yield parameters [83]. However, these approaches are not directly transferable to real-time applications. Therefore, we suggest that a more direct method, linking spectral and meteorological data to vegetation parameters, would be better suited for real-time tasks.

## 5. Conclusions

This work investigated the challenges posed by plastic film coverings in vineyards, and demonstrated that, despite interference, accurate estimation of crucial ground parameters was achieved; these included (i) ISR, which is strictly related to leaf area [72] and crop coefficients [37,84,85], and (ii) Ψstem, which is a reliable indicator of plant water status.

Preliminary analyses of the spectral time series confirmed the effects of the plastic sheets on the spectral responses of the vegetation. Therefore, a broad range of models, from simple linear regressions to more complex machine learning-based ones, were tested to identify the best model and the most important spectral/meteorological predictive features. Single linear regressions provided satisfactory results regarding ISR estimation; conversely, Ψstem appeared to require more complex and refined models. Specifically, Multivariate Regression, Random Forest Regression, and Support Vector Regression led to the best results.

The findings from this work suggest that Multiple Regression and machine learning models have high potential to generate more accurate predictions than ordinary regression methods, especially for agricultural applications where the relationships are multivariate and complex. However, on some occasions, even single bands or spectral indices can be used to monitor ISR. It is in fact evident that accurate machine learning-derived estimates required a relatively high number of predictors (features), suggesting a trade-off between model complexity and performance. This aspect has to be carefully considered and suggests the importance of a preliminary step of feature selection. However, it should be noted that a fairly simple feature selection method was employed and more effective ones like Genetic Algorithms (GAs) or Shapely Addictive Explanation (SHAP) should be considered in future studies. Additionally, recent studies investigated the potential of using time series-based Artificial Neural Networks for spectral index forecasting [86,87]. In this context, these forecast estimates could be employed to predict crop ecophysiological parameters and thus further enhance precision agriculture applications.

Finally, an approach was proposed to develop a method to assess the impact of the cover on the spectral signature at the beginning of the season. Further research is warranted to explore diverse types of plastic films, providing a comprehensive understanding of their influence on the spectral signature of vegetation, since the radiometric characteristics of plastic sheets have an impact on transmitted radiation [30,74,75] and can have a significant effect on the reflection from the canopies.

## Figures and Tables

**Table 1:** Phenological stages of grapevine cv Luisa (extended BBCH scale), with related DOY and GDD.

Principal Growth Stage	Description	BBCH Code	DOY	GDD
Sprouting	Bud burst: green shoot tips are clearly visible	09	100	24
Leaf development	Six leaves have unfolded	16	119	132
Inflorescence emergence	Inflorescence swelling, flowers closely pressed together	55	134	267
Flowering	80% of flowerhoods have fallen	68	141	360
Development of fruits	Fruit is set: fruits beginning to swell, remains of flowers are lost	71	145	425
	Berries are groat-sized, bunches beginning to hang	73	151	513
	Berries beginning to touch	77	162	668
	All berries are touching	79	181	998
Ripening of berries	Beginning of ripening: berries beginning to brighten in color	81	190	1153
	Berries brightening in color	83	207	1437
	Berries ripe for harvest	89	216	1593
Senescence	After harvest: end of wood maturation	91	234	1880

**Table 2:** S2 band technical features: central wavelength, bandwidth, ground sample distance (GSD), radiometric resolution, and temporal resolution [41].

Spectral Band	Central Wavelength (nm)	Band Width (nm)	GSD
B1 (Aerosol)	443	20	60
B2 (Blue)	490	65	10
B3 (Green)	560	35	10
B4 (Red)	665	30	10
B5 (Red Edge 5)	705	15	20
B6 (Red Edge 6)	740	15	20
B7 (Red Edge 7)	783	20	20
B8 (Near Infrared)	842	115	10
(B8A Near Infrared Plateau)	885	20	20
B9 (Water Vapor)	945	20	60
B10 (Cirrus)	1380	30	60
B11 (Short Wave Infrared 1)	1610	90	20
B12 (Short Wave Infrared 2)	2019	180	20
Radiometric resolution	12 bit		
Temporal resolution	5 days		

**Table 3:** Temporal resolution of Sentinel-2 images and ground data availability for 2022 from April to August (DOY: day of the year). First column reports the S2 images temporally closer to the ground surveys. Second column reports the S2 images used, either native or derived from the interpolation procedure (highlighted with an asterisk *).

	Closest Sentinel-2 Image	Sentinel-2 Image Used	ISR	Ψstem
DOY	117	119 *	119	-
	134	134	134	-
	142	141 *	141	-
	142	145 *	145	145
	152	151 *	151	151
	164	162 *	162	162
	184	181 *	181	181
	192	190 *	190	190
	202	201 *	201	201
	207	207	207	207
	217	216 *	216	216
	239	234 *	234	234

**Table 4:** Considered meteorological and spectral features.

Ecophysiological Parameters	Meteorological Feature	Spectral Features
ISR (%) Ψstem (Mpa)	GDD	B2-B3-B4-B5-B6-B7-B8-B8A-B11-B12
		NDVI-GNDVI-NDRE-EVI
		NDMI-NDWI-NDWI_2_-NDWI_3_

**Table 5:** Hyperparameter values tested to find the best RFR and SVR configuration.

ML Algorithm	Hyperparameters			
RFR	Trees: {50, 100, 200}	Maximum Depth: {None, 10, 20}	Minimum Sample Leaf: {1, 2, 4}	Maximum Features: {sqrt, log2, 1}
SVR	Kernel: {RBF}	C: {0.01, 0.1, 1, 10, 50, 100}	ε: {0.1, 0.2, 0.3, 0.5, 1, 2, 4}	γ: {scale, auto, 0.1, 0.5, 1, 2, 4, 10}

**Table 6:** Statistical description of ISR and Ψstem values collected on cv Luisa vines, on surveyed days of year (DOY) (SD = standard deviation).

DOY	ISR (%)				Ψstem (MPa)			
	Mean	Min	Max	SD	Mean	Min	Max	SD
119	24.86	18.78	30.32	4.15	-	-	-	-
134	38.86	33.27	43.54	3.73	-	-	-	-
141	44.16	38.33	49.37	3.70	-	-	-	-
145	52.05	44.65	59.13	4.01	−0.515	−0.605	−0.425	0.058
151	60.44	53.23	67.40	4.39	−0.569	−0.625	−0.485	0.039
162	72.94	57.55	80.78	7.35	−0.494	−0.550	−0.435	0.036
181	84.51	66.29	93.98	7.89	−0.617	−0.710	−0.490	0.066
190	87.11	74.39	93.57	5.11	−0.635	−0.740	−0.465	0.097
201	85.94	72.60	93.98	6.06	−1.031	−1.240	−0.875	0.120
207	83.40	72.13	90.95	6.12	−1.094	−1.335	−0.895	0.127
216	83.58	73.72	89.54	5.83	−0.837	−1.030	−0.700	0.112
234	87.48	78.14	93.70	4.87	−0.818	−1.090	−0.625	0.156

**Table 7:** Parameters and statistical coefficients of 1st-order polynomial linear regressions between ground ISR (%) and satellite features and of 2nd-order polynomial linear regression between ground ISR (%) and a meteorological feature (GDD). O-P intercept and O-P slope refer to the intercept and slope derived from the observed–predicted 1st-order polynomial linear model. RMSE, R^2^, O-P slope, and O-P intercept were computed from the test set derived from the cross-validation procedure.

Satellite Feature	Intercept	Slope	*p*-Value	R^2^	RMSE (%)	O-P Intercept	O-P Slope
B2	269	−1042	9.24 × 10^−23^	0.477	15.82	33.99	0.49
B3	278.8	−963	2.35 × 10^−20^	0.437	16.41	36.70	0.45
B4	203.5	−708.6	7.50 × 10^−41^	0.706	11.86	18.90	0.72
B5	281.9	−863.2	1.31 × 10^−27^	0.554	14.60	28.97	0.57
B6	−116.1	453.4	2.98 × 10^−66^	0.874	7.77	8.34	0.88
B7	−75.6	309.6	4.86 × 10^−72^	0.897	7.06	6.99	0.90
B8	−83.22	324.43	5.36 × 10^−66^	0.873	7.78	8.46	0.87
B8A	−82.07	311.56	2.20 × 10^−72^	0.897	7.02	6.90	0.90
B11	292.7	−740	3.63 × 10^−32^	0.614	13.59	25.16	0.62
B12	163.6	−568.2	5.53 × 10^−44^	0.737	11.21	17.32	0.74
NDRE	3.66	214.91	2.27 × 10^−65^	0.868	7.94	8.70	0.87
GNDVI	−14.59	233.37	2.18 × 10^−63^	0.857	8.25	9.27	0.86
NDVI	−5.79	179.19	1.30 × 10^−63^	0.860	8.19	9.18	0.86
NDMI	24.06	199.14	1.67 × 10^−67^	0.878	7.62	8.14	0.88
NDWI	−3.326	153.89	2.93 × 10^−61^	0.870	7.89	8.70	0.87
NDWI_2_	109.1	259.7	7.48 × 10^−4^	0.062	21.20	62.50	0.07
NDWI_3_	−84.24	82.38	5.73 × 10^−39^	0.756	10.81	16.27	0.76
EVI	4.01	108.30	1.45 × 10^−62^	0.856	8.29	9.47	0.86
GDD	13.85	a: −4 × 10^−7^ b: +0.001	3.4 × 10^−77^	0.917	6.31	5.53	0.91

**Table 8:** Statistical results of 1st-order polynomial regression relating ground-measured Ψstem (MPa) to satellite spectral features and meteorological data (GDD). O-P intercept and O-P slope refer to the intercept and slope derived from the observed–predicted 1st-order polynomial linear model. RMSE, R^2^, O-P slope, and O-P intercept were computed from the test set derived from the cross-validation procedure.

Satellite Feature	Intercept	Slope	*p*-Value	R^2^	RSME (MPa)	O-P Intercept	O-P Slope
B2	−2.675	10.250	2.01 × 10^−12^	0.357	0.183	−0.46	0.37
B3	−2.931	10.180	1.05 × 10^−14^	0.418	0.174	−0.41	0.43
B4	−1.760	5.617	2.45 × 10^−9^	0.270	0.195	−0.52	0.29
B5	−2.617	7.739	1.90 × 10^−12^	0.355	0.184	−0.46	0.38
B6	0.201	−2.193	1.67 × 10^−2^	0.041	0.224	−0.70	0.05
B7	0.227	−1.948	6.43 × 10^−4^	0.099	0.217	−0.66	0.10
B8	−0.069	−1.350	1.90 × 10^−2^	0.040	0.224	−0.70	0.04
B8A	0.413	−2.243	1.64 × 10^−4^	0.120	0.215	−0.64	0.12
B11	−2.526	6.051	6.88 × 10^−9^	0.255	0.197	−0.53	0.27
B12	−1.459	4.657	3.30 × 10^−8^	0.238	0.199	−0.55	0.25
NDRE	−0.202	−1.577	1.24 × 10^−6^	0.191	0.206	−0.59	0.20
GNDVI	0.012	−1.916	1.17 × 10^−7^	0.223	0.202	−0.56	0.23
NDVI	−0.134	−1.313	2.23 × 10^−6^	0.181	0.207	−0.59	0.19
NDMI	−0.264	−1.774	2.37 × 10^−7^	0.211	0.203	−0.57	0.22
NDWI	−0.092	−1.237	1.47 × 10^−6^	0.150	0.211	−0.61	0.16
NDWI_2_	−0.408	2.082	2.39 × 10^−2^	0.031	0.225	−0.70	0.04
NDWI_3_	0.452	−0.615	3.29 × 10^−7^	0.079	0.219	−0.67	0.08
EVI	−0.358	−0.565	7.94 × 10^−4^	0.093	0.218	−0.66	0.10
GDD	−0.371	−0.0003	8.81 × 10^−16^	0.435	0.172	−0.40	0.46

**Table 9:** Statistical results of MLR and MLRS approaches relating ground-measured ISR (%) and Ψstem (MPa) to satellite spectral features and meteorological data (GDD). O-P intercept and O-P slope refer to the intercept and slope derived from the observed–predicted 1st-order polynomial linear model. RMSE, R^2^, O-P slope, and O-P intercept were computed from the test set derived from the cross-validation procedure.

Method	Ecophysiological Parameter	GDD	Selected Features	RMSE	R^2^	O-P Intercept	O-P Slope
MLR	ISR	No	All	7.26%	0.885	5.951	0.911
		Yes	All	5.487%	0.932	4.633	0.932
MLRS	ISR	No	B8A, GNDVI	6.653%	0.898	6.564	0.902
		Yes	GDD, NDWI3, B7, NDWI, NDMI, NDVI	5.132%	0.944	3.049	0.953
MLR	Ψstem	No	All	0.134 MPa	0.555	−0.246	0.655
		Yes	All	0.136 MPa	0.527	−0.275	0.628
MLRS	Ψstem	No	B3, B8, NDRE, B4, GNDVI, B6, B7, B8A, NDVI	0.118 MPa	0.621	−0.254	0.660
		Yes	GDD, NDWI2, B8, NDMI, B4, NDRE, B7, B11	0.117 MPa	0.625	−0.251	0.657

**Table 10:** ML model parameters and predictors used to generate ISR estimates from the tuning step. O-P intercept and O-P slope refer to the intercept and slope derived from the observed–predicted 1st-order polynomial linear model. RMSE, R^2^, O-P slope, and O-P intercept were computed from the test set derived from the cross-validation procedure.

ML Algorithm	GDD	Best Hyperparameters	Selected Features	RMSE (%)	R^2^	O-P Intercept	O-P Slope
RFR	No	Trees: 50 Max Depth: 20 Min Leaves: 1 Max Features: log2	B2, B3, B4, B6, B7, B8, B8A, EVI, GNDVI, NDMI, NDWI_2_	5.1	0.95	4.72	0.94
SVR	No	Kernel: RBF C: 1 ε: 0.1 γ: scale	B6, B7	6.4	0.91	6.72	0.89
PLSR	No	—	B5, B8A	6.6	0.91	6.28	0.90
RFR	Yes	Trees: 100 Max Depth: 20 Min Leaves: 1 Max Features: log2	GDD, B2, B3, B4, B6, B8A, B12, NDMI, NDWI_2_	4.7	0.96	1.8	0.96
SVR	Yes	Kernel: RBF C: 100 ε: 0.1 γ: 0.1	GDD, B3, B7, B8, B11, B12, NDWI_2_	5.2	0.96	7.25	0.88
PLSR	Yes	—	GDD, B3, B7, B8A, B11, B12, EVI, GNDVI	5.0	0.95	4.20	0.94

**Table 11:** ML model parameters as predictors used to generate Ψstem estimates from the tuning step. O-P intercept and O-P slope refer to the intercept and slope derived from the observed–predicted 1st-order polynomial linear model. RMSE, R^2^, O-P slope, and O-P intercept were computed from the test set derived from the cross-validation procedure.

ML Algorithm	GDD	Best Hyperparameters	Selected Features	RMSE (MPa)	R^2^	O-P Intercept	O-P Slope
RFR	No	Trees: 50 Max Depth: 10 Min Leaves: 1 Max Features: SQRT	B3, B5, B8, NDWI	0.148	0.58	−0.30	0.58
SVR	No	Kernel: RBF C: 100 ε: 0.5 γ: 0.5	B3, B4, B5, B6, B7, B8A, NDRE, NDVI, NDWI_2_	0.125	0.71	−0.25	0.64
PLSR	No	—	B3, B4, B7, B8, B8A, NDRE	0.147	0.59	−0.28	0.62
RFR	Yes	Trees: 100 Max Depth: 10 Min Leaves: 1 Max Features: sqrt	GDD, B4, B8A, EVI	0.101	0.81	−0.17	0.76
SVR	Yes	Kernel: RBF C: 50 ε: 0.1 γ: 4	GDD, B4, B6, B12, NDMI	0.122	0.72	−0.22	0.69
PLSR	Yes	—	GDD, B8, B8A, EVI, NDRE	0.136	0.65	−0.25	0.66

## Data Availability

The datasets presented in this article are not readily available because the data are part of an ongoing study. Requests to access the datasets should be directed to Alessandro Farbo (alessandro.farbo@unito.it).

## References

[1] Global Grape Production in 2022 Reached the Second Highest Peak of the Last Twenty Years—Wine Industry Advisor. https://winetitles.com.au/global-grape-production-in-2022-reached-the-second-highest-peak-of-the-last-twenty-years/.

[2] Marín D., Armengol J., Carbonell-Bejerano P., Escalona J.M., Gramaje D., Hernández-Montes E., Intrigliolo D.S., Martínez-Zapater J.M., Medrano H., Mirás-Avalos J.M. (2021). Challenges of Viticulture Adaptation to Global Change: Tackling the Issue from the Roots. Aust. J. Grape Wine Res..

[3] Matese A., Filippo Di Gennaro S. (2015). Technology in Precision Viticulture: A State of the Art Review. Int. J. Wine Res..

[4] Šimanský V., Wójcik-Gront E., Jonczak J., Horák J. (2023). Optimizing Soil Management for Sustainable Viticulture: Insights from a Rendzic Leptosol Vineyard in the Nitra Wine Region, Slovakia. Agronomy.

[5] Ferro M.V., Catania P. (2023). Technologies and Innovative Methods for Precision Viticulture: A Comprehensive Review. Horticulturae.

[6] Laroche-Pinel E., Albughdadi M., Duthoit S., Chéret V., Rousseau J., Clenet H. (2021). Understanding Vine Hyperspectral Signature through Different Irrigation Plans: A First Step to Monitor Vineyard Water Status. Remote Sens..

[7] Cohen Y., Gogumalla P., Bahat I., Netzer Y., Ben-Gal A., Lenski I., Michael Y., Helman D. (2019). Can Time Series of Multispectral Satellite Images Be Used to Estimate Stem Water Potential in Vineyards?. Precision Agriculture ’19.

[8] Ali A., Imran M. (2020). Evaluating the Potential of Red Edge Position (REP) of Hyperspectral Remote Sensing Data for Real Time Estimation of LAI & Chlorophyll Content of Kinnow Mandarin (*Citrus Reticulata*) Fruit Orchards. Sci. Hortic..

[9] Kalisperakis I., Stentoumis C., Grammatikopoulos L., Karantzalos K. Leaf Area Index Estimation in Vineyards from Uav Hyperspectral Data, 2D Image Mosaics and 3D Canopy Surface Models. Proceedings of the International Archives of the Photogrammetry, Remote Sensing and Spatial Information Sciences—ISPRS Archives.

[10] Rozenstein O., Haymann N., Kaplan G., Tanny J. (2018). Estimating Cotton Water Consumption Using a Time Series of Sentinel-2 Imagery. Agric. Water Manag..

[11] Rozenstein O., Haymann N., Kaplan G., Tanny J. (2019). Validation of the Cotton Crop Coefficient Estimation Model Based on Sentinel-2 Imagery and Eddy Covariance Measurements. Agric. Water Manag..

[12] Ramoelo A., Cho M.A. (2018). Explaining Leaf Nitrogen Distribution in a Semi-Arid Environment Predicted on Sentinel-2 Imagery Using a Field Spectroscopy Derived Model. Remote Sens..

[13] Peng X., Chen D., Zhou Z., Zhang Z., Xu C., Zha Q., Wang F., Hu X. (2022). Prediction of the Nitrogen, Phosphorus and Potassium Contents in Grape Leaves at Different Growth Stages Based on UAV Multispectral Remote Sensing. Remote Sens..

[14] Jesus J., Santos F., Gomes A., Teodoro A.C. (2020). Temporal Analysis of the Vineyard Phenology from Remote Sensing Data Using Google Earth Engine. Proceedings of the Remote Sensing for Agriculture, Ecosystems, and Hydrology XXII.

[15] Fraga H., Amraoui M., Malheiro A.C., Moutinho-Pereira J., Eiras-Dias J., Silvestre J., Santos J.A. (2014). Examining the Relationship between the Enhanced Vegetation Index and Grapevine Phenology. Eur. J. Remote Sens..

[16] Laroche-Pinel E., Duthoit S., Albughdadi M., Costard A.D., Rousseau J., Chéret V., Clenet H. (2021). Towards Vine Water Status Monitoring on a Large Scale Using Sentinel-2 Images. Remote Sens..

[17] Ilniyaz O., Du Q., Shen H., He W., Feng L., Azadi H., Kurban A., Chen X. (2023). Leaf Area Index Estimation of Pergola-Trained Vineyards in Arid Regions Using Classical and Deep Learning Methods Based on UAV-Based RGB Images. Comput. Electron. Agric..

[18] Orusa T., Borgogno Mondino E. (2021). Exploring Short-Term Climate Change Effects on Rangelands and Broad-Leaved Forests by Free Satellite Data in Aosta Valley (Northwest Italy). Climate.

[19] Chen X., Chen J., Jia X., Wu J. Impact of Collinearity on Linear and Nonlinear Spectral Mixture Analysis. Proceedings of the 2010 2nd Workshop on Hyperspectral Image and Signal Processing: Evolution in Remote Sensing.

[20] Lopez-Fornieles E., Brunel G., Rancon F., Gaci B., Metz M., Devaux N., Taylor J., Tisseyre B., Roger J.-M. (2022). Potential of Multiway PLS (N-PLS) Regression Method to Analyse Time-Series of Multispectral Images: A Case Study in Agriculture. Remote Sens..

[21] Yu S., Fan J., Lu X., Wen W., Shao S., Liang D., Yang X., Guo X., Zhao C. (2023). Deep Learning Models Based on Hyperspectral Data and Time-Series Phenotypes for Predicting Quality Attributes in Lettuces under Water Stress. Comput. Electron. Agric..

[22] Loggenberg K., Strever A., Greyling B., Poona N. (2018). Modelling Water Stress in a Shiraz Vineyard Using Hyperspectral Imaging and Machine Learning. Remote Sens..

[23] Doktor D., Lausch A., Spengler D., Thurner M. (2014). Extraction of Plant Physiological Status from Hyperspectral Signatures Using Machine Learning Methods. Remote Sens..

[24] Ilniyaz O., Kurban A., Du Q. (2022). Leaf Area Index Estimation of Pergola-Trained Vineyards in Arid Regions Based on UAV RGB and Multispectral Data Using Machine Learning Methods. Remote Sens..

[25] Suwanlee S.R., Pinasu D., Som-ard J., Borgogno-Mondino E., Sarvia F. (2024). Estimating Sugarcane Aboveground Biomass and Carbon Stock Using the Combined Time Series of Sentinel Data with Machine Learning Algorithms. Remote Sens..

[26] Novello V., de Palma L. (2008). Growing Grapes under Cover. Acta Hortic..

[27] Novello V., de Palma L., Tarricone L., Vox G. (2000). Effects of Different Plastic Sheet Coverings on Microclimate and Berry Ripening of Table Grape Cv “Matilde”. OENO One.

[28] Fidelibus M.W., Vasquez S.J., Kurtural S.K. (2016). Late-Season Plastic Canopy Covers Affect Canopy Microclimate and Fruit Quality of ‘Autumn King’ and ‘Redglobe’ Table Grapes. HortTechnology.

[29] Borgogno-Mondino E., de Palma L., Novello V. (2020). Investigating Sentinel 2 Multispectral Imagery Efficiency in Describing Spectral Response of Vineyards Covered with Plastic Sheets. Agronomy.

[30] Vox G., Schettini E., Scarascia Mugnozza G., Tarricone L., de Palma L. (2014). Covering Plastic Films for Vineyard Protected Cultivation. Acta Hortic..

[31] Kittas C., Rigakis N., Katsoulas N., Bartzanas T. (2009). Influence of Shading Screens on Microclimate, Growth and Productivity of Tomato. Acta Hortic..

[32] Borgogno-Mondino E., Lessio A., Tarricone L., Novello V., de Palma L. (2018). A Comparison between Multispectral Aerial and Satellite Imagery in Precision Viticulture. Precis. Agric..

[33] Beck H.E., Zimmermann N.E., McVicar T.R., Vergopolan N., Berg A., Wood E.F. (2018). Present and Future Köppen-Geiger Climate Classification Maps at 1-Km Resolution. Sci. Data.

[34] Mcmaster G. (1997). Growing Degree-Days: One Equation, Two Interpretations. Agric. For. Meteorol..

[35] Winkler A.J., Cook J.A., Kliewer W.M., Lider L.A., Cerruti L. (1974). General Viticulture.

[36] Lorenz D.H., Eichhorn K.W., Bleiholder H., Klose R., Meier U., Weber E. (1995). Growth Stages of the Grapevine: Phenological Growth Stages of the Grapevine (*Vitis vinifera* L. ssp. Vinifera)—Codes and Descriptions According to the Extended BBCH Scale†. Aust. J. Grape Wine Res..

[37] Villagra P., García de Cortázar V., Ferreyra R., Aspillaga C., Zúñiga C., Ortega-Farias S., Sellés G. (2014). Estimation of Water Requirements and Kc Values of &rsquo;Thompson Seedless&rsquo; Table Grapes Grown in the Overhead Trellis System, Using the Eddy Covariance Method. Chil. J. Agric. Res..

[38] Choné X., Van Leeuwen C., Dubourdieu D., Gaudillère J.P. (2001). Stem Water Potential Is a Sensitive Indicator of Grapevine Water Status. Ann. Bot..

[39] Dardanelli G., Maltese A., Pipitone C., Pisciotta A., Lo Brutto M. (2021). NRTK, PPP or Static, That Is the Question. Testing Different Positioning Solutions for GNSS Survey. Remote Sens..

[40] Gascon F., Bouzinac C., Thépaut O., Jung M., Francesconi B., Louis J., Lonjou V., Lafrance B., Massera S., Gaudel-Vacaresse A. (2017). Copernicus Sentinel-2A Calibration and Products Validation Status. Remote Sens..

[41] Sentinel-2-Missions-Sentinel. https://copernicus.eu/missions/sentinel-2.

[42] David R.M., Rosser N.J., Donoghue D.N.M. (2022). Improving above Ground Biomass Estimates of Southern Africa Dryland Forests by Combining Sentinel-1 SAR and Sentinel-2 Multispectral Imagery. Remote Sens. Environ..

[43] Borgogno-Mondino E., Farbo A., Novello V., Palma L. (2022). de A Fast Regression-Based Approach to Map Water Status of Pomegranate Orchards with Sentinel 2 Data. Horticulturae.

[44] Petersen L.K. (2018). Real-Time Prediction of Crop Yields From MODIS Relative Vegetation Health: A Continent-Wide Analysis of Africa. Remote Sens..

[45] Farbo A., Sarvia F., De Petris S., Borgogno-Mondino E. Preliminary Concerns about Agronomic Interpretation of Ndvi Time Series from Sentinel-2 Data: Phenology and Thermal Efficiency of Winter Wheat in Piemonte (Nw Italy). Proceedings of the The International Archives of Photogrammetry, Remote Sensing and Spatial Information Sciences.

[46] Torgbor B.A., Rahman M.M., Robson A., Brinkhoff J., Khan A. (2022). Assessing the Potential of Sentinel-2 Derived Vegetation Indices to Retrieve Phenological Stages of Mango in Ghana. Horticulturae.

[47] Gao B.-C. (1996). NDWI—A Normalized Difference Water Index for Remote Sensing of Vegetation Liquid Water from Space. Remote Sens. Environ..

[48] Szabó S., Gácsi Z., Balázs B. (2016). Specific Features of NDVI, NDWI and MNDWI as Reflected in Land Cover Categories. Acta Geogr. Debrecina Landsc. Environ. Ser..

[49] Masina M., Lambertini A., Daprà I., Mandanici E., Lamberti A. (2020). Remote Sensing Analysis of Surface Temperature from Heterogeneous Data in a Maize Field and Related Water Stress. Remote Sens..

[50] Pan Z., Hu Y., Cao B. (2017). Construction of Smooth Daily Remote Sensing Time Series Data: A Higher Spatiotemporal Resolution Perspective. Open Geospat. Data Softw. Stand..

[51] Rybski D., Neumann J., Kropp J., Schellnhuber H.-J. (2011). A Review on the Pettitt Test Pettitt-Test. Extremis: Disruptive Events and Trends in Climate and Hydrology.

[52] Romero M., Luo Y., Su B., Fuentes S. (2018). Vineyard Water Status Estimation Using Multispectral Imagery from an UAV Platform and Machine Learning Algorithms for Irrigation Scheduling Management. Comput. Electron. Agric..

[53] Nikolenko S.I. (2021). Synthetic Data for Deep Learning.

[54] Bejani M.M., Ghatee M. (2021). A Systematic Review on Overfitting Control in Shallow and Deep Neural Networks. Artif. Intell. Rev..

[55] Kuhn M., Johnson K. (2019). Feature Engineering and Selection: A Practical Approach for Predictive Models.

[56] Bargagli Stoffi F.J., Cevolani G., Gnecco G. (2022). Simple Models in Complex Worlds: Occam’s Razor and Statistical Learning Theory. Minds Mach..

[57] Everitt B. (1992). Book Reviews: Chambers JM, Hastie TJ Eds 1992: Statisti Cal Models in S. California: Wadsworth and Brooks/Cole. Stat. Methods Med. Res..

[58] Darvishzadeh R., Skidmore A., Schlerf M., Atzberger C., Corsi F., Cho M. (2008). LAI and Chlorophyll Estimation for a Heterogeneous Grassland Using Hyperspectral Measurements. ISPRS J. Photogramm. Remote Sens..

[59] Aho K., Derryberry D., Peterson T. (2014). Model Selection for Ecologists: The Worldviews of AIC and BIC. Ecology.

[60] Otgonbayar M., Atzberger C., Chambers J., Damdinsuren A. (2019). Mapping Pasture Biomass in Mongolia Using Partial Least Squares, Random Forest Regression and Landsat 8 Imagery. Int. J. Remote Sens..

[61] Belgiu M., Drăguţ L. (2016). Random Forest in Remote Sensing: A Review of Applications and Future Directions. ISPRS J. Photogramm. Remote Sens..

[62] Mountrakis G., Im J., Ogole C. (2011). Support Vector Machines in Remote Sensing: A Review. ISPRS J. Photogramm. Remote Sens..

[63] Willmott C.J. (1981). On the Validation of Models. Phys. Geogr..

[64] R: The R Project for Statistical Computing. https://www.r-project.org/.

[65] van Leeuwen C., Trégoat O., Choné X., Bois B., Pernet D., Gaudillère J.-P. (2009). Vine Water Status Is a Key Factor in Grape Ripening and Vintage Quality for Red Bordeaux Wine. How Can It Be Assessed for Vineyard Management Purposes?. OENO One.

[66] Giovos R., Tassopoulos D., Kalivas D., Lougkos N., Priovolou A. (2021). Remote Sensing Vegetation Indices in Viticulture: A Critical Review. Agriculture.

[67] Stolarski O., Fraga H., Sousa J.J., Pádua L. (2022). Synergistic Use of Sentinel-2 and UAV Multispectral Data to Improve and Optimize Viticulture Management. Drones.

[68] Tassopoulos D., Kalivas D., Giovos R., Lougkos N., Priovolou A. (2021). Sentinel-2 Imagery Monitoring Vine Growth Related to Topography in a Protected Designation of Origin Region. Agriculture.

[69] Nonni F., Malacarne D., Pappalardo S.E., Codato D., Meggio F., Marchi M.D. (2018). Sentinel-2 Data Analysis and Comparison with UAV Multispectral Images for Precision Viticulture. GI_Forum 2018.

[70] Comparetti A., Marques da Silva J.R. (2022). Use of Sentinel-2 Satellite for Spatially Variable Rate Fertiliser Management in a Sicilian Vineyard. Sustainability.

[71] Mirás-Avalos J.M., Araujo E.S. (2021). Optimization of Vineyard Water Management: Challenges, Strategies, and Perspectives. Water.

[72] López-Lozano R., Casterad M.A. (2013). Comparison of Different Protocols for Indirect Measurement of Leaf Area Index with Ceptometers in Vertically Trained Vineyards. Aust. J. Grape Wine Res..

[73] Weiss M., Baret F., Smith G.J., Jonckheere I., Coppin P. (2004). Review of Methods for in Situ Leaf Area Index (LAI) Determination: Part II. Estimation of LAI, Errors and Sampling. Agric. For. Meteorol..

[74] de Palma L., Vox G., Schettini E., Novello V. (2022). Reduction of Evapotranspiration in Microenvironment Conditions of Table Grape Vineyards Protected by Different Types of Plastic Covers. Agronomy.

[75] Vox G., Scarascia Mugnozza G., Schettini E., de Palma L., Tarricone L., Gentilesco G., Vitali M. (2012). Radiometric Properties of Plastic Films for Vineyard Covering and Their Influence on Vine Physiology and Production. Acta Hortic..

[76] Kang Y., Gao F., Anderson M., Kustas W., Nieto H., Knipper K., Yang Y., White W., Alfieri J., Torres-Rua A. (2022). Evaluation of Satellite Leaf Area Index in California Vineyards for Improving Water Use Estimation. Irrig. Sci..

[77] Vélez S., Barajas E., Rubio J.A., Vacas R., Poblete-Echeverría C. (2020). Effect of Missing Vines on Total Leaf Area Determined by NDVI Calculated from Sentinel Satellite Data: Progressive Vine Removal Experiments. Appl. Sci..

[78] Garofalo S.P., Giannico V., Costanza L., Alhajj Ali S., Camposeo S., Lopriore G., Pedrero Salcedo F., Vivaldi G.A. (2024). Prediction of Stem Water Potential in Olive Orchards Using High-Resolution Planet Satellite Images and Machine Learning Techniques. Agronomy.

[79] Rapaport T., Hochberg U., Shoshany M., Karnieli A., Rachmilevitch S. (2015). Combining Leaf Physiology, Hyperspectral Imaging and Partial Least Squares-Regression (PLS-R) for Grapevine Water Status Assessment. ISPRS J. Photogramm. Remote Sens..

[80] Lin Y., Zhu Z., Guo W., Sun Y., Yang X., Kovalskyy V. (2020). Continuous Monitoring of Cotton Stem Water Potential Using Sentinel-2 Imagery. Remote Sens..

[81] Caruso G., Palai G. (2023). Assessing Grapevine Water Status Using Sentinel-2 Images. Italus Hortus.

[82] Pôças I., Gonçalves J., Costa P.M., Gonçalves I., Pereira L.S., Cunha M. (2017). Hyperspectral-Based Predictive Modelling of Grapevine Water Status in the Portuguese Douro Wine Region. Int. J. Appl. Earth Obs. Geoinf..

[83] Vélez S., Rançon F., Barajas E., Brunel G., Rubio J.A., Tisseyre B. (2022). Potential of Functional Analysis Applied to Sentinel-2 Time-Series to Assess Relevant Agronomic Parameters at the within-Field Level in Viticulture. Comput. Electron. Agric..

[84] Ayars J.E., Johnson R.S., Phene C.J., Trout T.J., Clark D.A., Mead R.M. (2003). Water Use by Drip-Irrigated Late-Season Peaches. Irrig. Sci..

[85] Picón-Toro J., González-Dugo V., Uriarte D., Mancha L.A., Testi L. (2012). Effects of Canopy Size and Water Stress over the Crop Coefficient of a “Tempranillo” Vineyard in South-Western Spain. Irrig. Sci..

[86] Farbo A., Sarvia F., De Petris S., Basile V., Borgogno-Mondino E. (2024). Forecasting Corn NDVI through AI-Based Approaches Using Sentinel 2 Image Time Series. ISPRS J. Photogramm. Remote Sens..

[87] Cavalli S., Penzotti G., Amoretti M., Caselli S. (2023). A Machine Learning Approach for NDVI Forecasting Based on Sentinel-2 Data.

